# Proanthocyanidins: novel treatment for psoriasis that reduces oxidative stress and modulates Th17 and Treg cells

**DOI:** 10.1080/13510002.2018.1462027

**Published:** 2018-04-09

**Authors:** Rui Lai, Dehai Xian, Xia Xiong, Lingyu Yang, Jing Song, Jianqiao Zhong

**Affiliations:** a Department of Dermatology, the Affiliated Hospital of Southwest Medical University, Luzhou, People's Republic of China; b Department of Anatomy, Southwest Medical University, Luzhou, People's Republic of China

**Keywords:** Proanthocyanidins, psoriasis, oxidative stress, T cells

## Abstract

Psoriasis is a common, chronic, inflammatory skin disease that affects 2%–4% of the global population. Recent studies have shown that increased oxidative stress (OS) and T-cell abnormalities are central to the pathogenesis of this disease. The resulting reactive oxygen species (ROS) induces proliferation and differentiation of Th17/Th1/Th22 cells and inhibits the anti-inflammatory activities of regulatory T lymphocytes (Treg). Subsequent secretions of inflammatory cytokines, such as interleukin (IL)-17, IL-22, tumor necrosis factor alpha (TNF-α), and interferon-gamma (IFN-γ), and vascular endothelial growth factor (VEGF), stimulate keratinocyte proliferation and angiogenesis.

Proanthocyanidins are a class of flavonoids from plants and fruits, and have various antioxidant, anti-inflammatory, and anti-angiogenic properties. Numerous reports have demonstrated therapeutic effects of proanthocyanidins for various diseases. Among clinical activities, proanthocyanidins suppress cell proliferation, prevent OS, and regulate Th17/Treg cells. Because the pathogenesis of psoriasis involves OS and T cells dysregulation, we reviewed the effects of proanthocyanidins on OS, Th17 and Treg cell activities, and keratinocyte proliferation and angiogenesis. Data from multiple previous studies warrant consideration of proanthocyanidins as a promising strategy for the treatment of psoriasis.

## Introduction

Psoriasis, a chronic immune-mediated inflammatory relapsing skin disorder, is characterized by epidermal hyperplasia, angiogenesis, and inflammatory cells infiltration [1]. Psoriasis currently affects 2%–4% of the global population and impacts quality of life by causing physical and psychological trauma [2,3]. Although the etiology of psoriasis remains unclear, it is widely considered that oxidative stress (OS) and T-cell dysregulation are the key pathogenic factors. Various treatments have been utilized to treat psoriasis, including topical preparations containing corticosteroids, retinoid derivatives, synthetic vitamin D3 analogs, tar, or anthralin; systemic medications, such as immunosuppressive agents and calcineurin inhibitors acitretin and isotretinoin; and photochemotherapy (PUVA) and UVB irradiation [4–6]. However, these therapies have transient curative effects and hardly prevent relapse. Moreover, most psoriasis therapies are unsuitable for long-term use due to considerable side effects and high costs [7,8]. Consequently, a long-term cure for psoriasis is eagerly awaited.

Recently, studies have shown that natural proanthocyanidins have powerful antioxidant, anti-inflammatory, immunosuppressive, anti-angiogenic, and anti-proliferative activities, and have no adverse effects [9,10]. Proanthocyanidins are polyphenols from various plants and fruits, and are present at high levels in grape seeds, cranberries, red wine, metasequoia, and glyptostroboides. Increasing evidence indicates that proanthocyanidins offer effective and safe treatments for various diseases, including cardiovascular disease, diabetes, autoimmune arthritis, and squamous cell carcinoma, primarily by ameliorating OS, regulating cell differentiation, and inhibiting cell proliferation. However, few studies demonstrate the efficacy of proanthocyanidins in the treatment of psoriasis [11–16]. Because psoriasis is an immune-mediated, inflammatory disease that leads to OS and T-cell abnormalities, we reviewed the evidence of treatment potential of psoriasis.

## Pathogenesis of psoriasis

Genetic, environmental, and immunological factors have been considered in connection with the etiology of psoriasis [17,18], and an increasing number of studies have demonstrated that OS and immune inflammation are central to the pathogenesis of psoriasis. Although several proinflammatory factors and cytokines have been implicated, OS is caused by endogenous and exogenous factors and contributes to increased levels of reactive oxygen species (ROS), which initially triggers T-cell imbalances and inflammatory reactions, and then promotes the release of inflammatory cytokines that stimulate keratinocyte proliferation and angiogenesis [19,20]. These molecular and histopathological alterations have been implicated in clinical manifestations of psoriasis [21–23], with increased ROS and dysregulated T cells being central.

Redox imbalances are increasingly implicated in the pathogenesis of psoriasis and manifest throughout the disease. In particular, multiple studies have shown significant aberrations of OS parameters in psoriasis patients [24–26], and most show marked decreases in the antioxidant enzymes catalase (CAT), superoxide dismutase (SOD), and glutathione peroxidase (GSH-Px) in psoriatic lesions and in matched serum samples. Conversely, increased levels of malondialdehyde (MDA), nitric oxide (NO), superoxide radical (${\text{O}}_{2}^{-}$), and inducible nitric oxide synthase (iNOS), have been found in psoriatic lesions [27–29]. Most of these OS biomarkers are closely related to the severity and progression of psoriasis. Previous studies show that OS, predominantly related to increased ROS, has significant effect on T lymphocytes, dendritic cells (DCs), and keratinocytes, and on inflammatory signaling and angiogenesis [30,31]. By stimulating several proinflammatory signaling pathways including nuclear factor kappa B (NF-κB), mitogen activated protein kinase (MAPK), and the Janus kinase–signal transducers and activators of transcription (JAK-STAT), increased ROS elicit the release of proinflammatory mediators and secretions of vascular endothelial growth factor (VEGF), which induces angiogenesis. Concomitantly, T helper (Th)1/Th17 cells are activated and regulatory T (Treg) cells are inhibited [32–34]. Th1 activation may induce occurrence of psoriasis, while Th17 cells, the most central factor of psoriasis, facilitate further development of psoriasis via production of several inflammatory cytokines and stimulation of neutrophil and macrophage infiltration [35,36]. Consequently, Th1/Th17 cells interact with DCs, mast cells, macrophages, and neutrophils to coordinate inflammatory responses involving interleukin (IL)-8, IL-12, IL-17, IL-19, IL-22, IL-23, tumor necrosis factor alpha (TNF-α), transforming growth factor-β (TGF-β), and interferon-gamma (IFN-γ) [37,38]. Via ROS-mediated transcription factors and proliferation pathways, these cytokines promote T-cell and keratinocyte proliferation and differentiation, and induce the above signals to mediate the immunopathological process of psoriasis [39,40].

**Figure 1.**
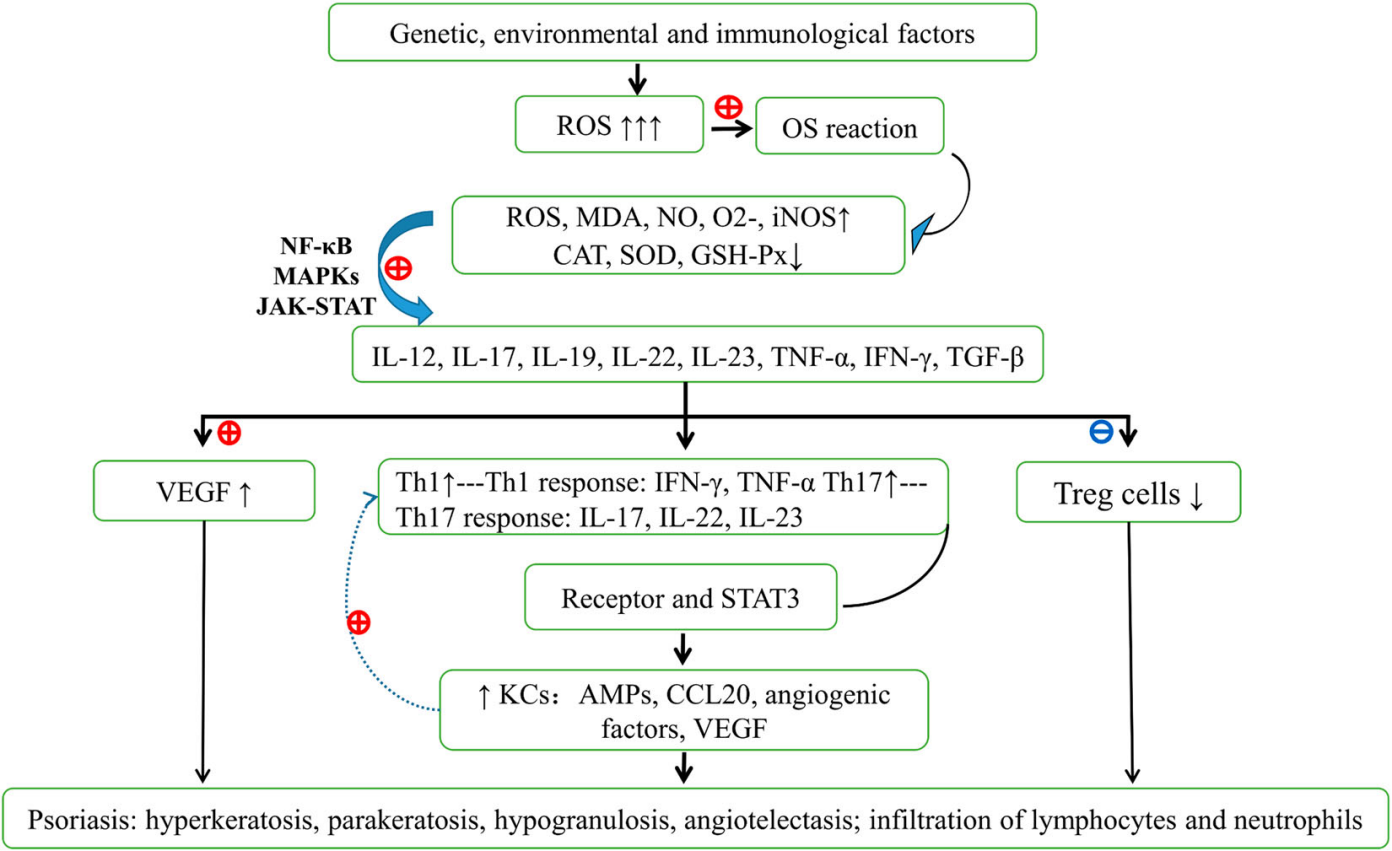
The primary pathogenesis of psoriasis. Genetic, environmental, and immunological factors induce active oxidative stress (OS) responses, leading to increased reactive oxygen species (ROS), malodialdehyde, nitric oxide, superoxide, inducible nitric oxide synthase, and decreased catalase, superoxide dismutase, and glutathione peroxidase in psoriatic lesions and in serum. Abnormal OS follows excessive ROS production, active dendritic cells, mast cells, macrophages and neutrophils, and activated nuclear factor kappa B, mitogen activated protein kinase, and Janus kinase-signal transducers and activators of transcription signaling pathways. Under these conditions, cells secrete proinflammatory cytokines, including IL-12, 17, 19, 22, 23, tumor necrosis factor alpha (TNF-α), interferon-gamma (IFN-γ), and transforming growth factor beta. Released cytokines promote the expression of vascular endothelial growth factor and encourage Th1/Th17 cell activation and decreased Treg cell activity. Increased numbers of Th1 and Th17 cells secrete IFN-γ, TNF-α, IL-17, IL-21, IL-22, IL-23, and IL-26, and subsequent STAT3 signaling leads to increased keratinocyte numbers, thus contributing to psoriasis. OS, oxidative stress; ROS, reactive oxygen species; NO, nitric oxide; MDA, malondialdehyde; ${\text{O}}_{2}^{-}$, superoxide radical; SOD, superoxide dismutase; GSH-PX, glutathione peroxidase; CAT, catalase; DCs, dendritic cells; IL, interleukin; TNF-α, tumor necrosis factor alpha; TGF-β, transforming growth factor beta; IFN-γ, interferon-gamma; STAT3, signal transducers and activators of transcription; Th, T helper; Treg cells, regulatory T cells; VEGF, vascular endothelial growth factor; KCs, keratinocytes.

Psoriasis, as a disease of multifactorial involvement, is also implicated in inherited susceptibility alleles, TNF-α gene polymorphism in particular. TNF-α is generally considered as the master proinflammatory cytokine and is deemed to be a key candidate gene for the pathogenesis of psoriasis, which can accelerate the infiltration of lymphocytes, neutrophils and monocytes. The single nucleotide polymorphisms of TNF-α at loci + 489 GG and GA, −308 G/G, −238 and −857C/T and + 489 are strongly associated with psoriasis and psoriasis arthropathica and may become a vital pharmaceutical therapy target for these conditions [41]. Although TNF-α has been investigated in multiple studies of psoriasis, the cytokines IL-12, IL-17, IL-19, IL-22, and IL-23 also play central roles. In particular, IL-12 and IL-23 induce Th1 and Th17-biased immune responses; IL-23 dominates Th17 activation, proliferation and maintenance [22,39,42], while IL-12 polarizes Th1 responses, leading to the production of Th17 (IL-17, IL-22, and IL-23) and Th1 cytokines (IFN-γ and TNF-α), respectively [43,44]. Th17 cells and Treg cells also represent two CD4(+) T-cell subsets, and are central players in the pathogenesis of psoriasis. Ratio of Th17 to Treg cells increase in psoriasis patients and is positively correlated with disease severity [45]. Th17 cells are derived from CD4+ T cells in the presence of IL-6, IL-23, and TGF-β, which play a central role in the chronic inflammatory diseases (psoriasis in particular) and are considered responsible for the chronic course of psoriasis [21,31,35]. These cell types secrete inflammatory cytokines such as IL-6, IL-17, IL-21, IL-22, IL-23, IL-26, and TNF-α (IL-17 in particular); IL-17 not only promotes keratinocytes proliferation but also encourages the production of intercellular adhesion molecule-1, IL-6, IL-1, IL-8, prostaglandin E2, TNF-α and IFN-γ [35]. The above processes provoke and exacerbate the immune responses and contribute to sustained psoriatic inflammation [22,31,42]. In contrast, CD4+ T cells differentiate into Treg cells in the presence of TGF-β, and subsequently express Forkhead Box P3 (Foxp3) [46]. Foxp3 (+) Treg cells have prominent functions in the maintenance of immunological tolerance to self-antigens, in the counteraction of inflammatory activity of effector Th cells, and in the regulation of Th17 differentiation [22]. However, during the onset of psoriasis, the anti-inflammatory effects of Treg cells against T-cell proliferation and IFN-γ secretion are impaired, and number of Treg cells is reduced [47]. Notably, many of the cytokines produced by activated Th17 cells induce keratinocyte proliferation in psoriasis patients, and following receptor binding and downstream signaling via STAT3, IL-23 contributes to the development of psoriasis [48,49]. The STAT3 pathway has been widely associated with proliferation and is markedly active in psoriasis patients, likely leading to an increased IL-17 expression [50]. Persistent activation of STAT3 leads to increased Th17 and keratinocyte proliferation [49]. Moreover, following stimulation of STAT3 phosphorylation by IL-6/IL-22, the ensuing signaling pathway leads to overexpression of VEGF during psoriasis. Although IL-17 is widely associated with psoriatic keratinocyte proliferation and prosoplasia [51–54], activated keratinocytes produce numerous cytokines and chemokines, including adenosine monophosphates (AMPs), angiogenic factors, and CCL20, which subsequently activate T cells, recruit neutrophils, and form a sustaining and amplifying inflammation loop [55]. Due to these molecular and cellular alterations, histopathologic features of psoriasis ultimately present as hyperkeratosis, parakeratosis, hypogranulosis, angiogenesis of dermal papillae, and sustained infiltration of lymphocytes and neutrophils [56]. The pathogenic mechanisms of psoriasis are summarized in Figure 1.

## Characteristics and clinical applications of proanthocyanidins

Proanthocyanidins belong to plant flavonoids, including catechin and epicatechin, and have antioxidant, anti-inflammatory, anti-angiogenesic, anti-proliferative, and immunomodulatory effects [16,57–65]. Proanthocyanidins have been isolated from grapes, apples, metasequoia bark, cinnamon, aronia fruit, cocoa beans, bilberry, cranberry, black currant, and various other plants [16,66]. As powerful antioxidants and free-radical scavengers, proanthocyanidins have a wide range of application in the treatment for various OS-related complaints [67]. Previous studies have shown that proanthocyanidins antagonize OS-mediated damage and enforce antioxidant capacity by modulating several signaling pathways, eliminating ROS and MDA, and upregulating antioxidants or detoxication enzymes, including hemeoxygenase-1(HO-1), CAT, SOD, and GSH-Px [14,57,58,68]. Mantena *et al.* found that dietary proanthocyanidin supplements help to prevent UV-induced skin disorders by scavenging hydroxyl radicals and superoxide anions, and by enhancing CAT, SOD, GSH, and GSH-Px activities [68]. Similarly, Som *et al.* reported that proanthocyanidins protect skin from UVB-induced damage by inhibiting MAPK/NF-κB signaling pathways [57]. Moreover, findings from Sun [58] and Miao *et al.* [14] show elevated Nrf2 and HO-1 protein expression in the presence of proanthocyanidins. HO-1 in particular reportedly blocks the STAT3 signaling pathway [53] and further mitigates oxidative damage in diabetes, following induction by zearalenone.

Given the number of demonstrated effects on various markers of inflammation and immune abnormalities, proanthocyanidins potently regulate T cells and inflammatory cytokines and have a high potential as treatments for inflammatory and autoimmune diseases. In a well-established autoimmune arthritis mice model, proanthocyanidins alleviated collagen-induced arthritis symptoms of mice by reducing Th17 cell numbers, increasing Treg cell numbers, and suppressing the release of the STAT3-induced cytokines IL-21, IL-22, IL-26, and IL-17 [15]. Concomitantly, Chen treated allergic contact dermatitis with proanthocyanidins and observed direct inhibition of Th cell activation and significant reductions in Th17 cytokines (IL-2, IFN-γ, and IL-17) expression levels [59]. Proanthocyanidins also inhibit LPS-induced inflammation via inhibiting the mRNA expression of TNF-α and IL-1β and suppressing MAPK and NF-κB signal pathways [60]. Moreover, related studies demonstrated strong anti-proliferative properties of proanthocyanidins in squamous carcinoma cells, with increased apoptosis and autophagy [16,61]. Furthermore, proanthocyanidins were shown to be excellent inhibitors of VEGF and had *in vitro* and *in vivo* anti-angiogenic properties that affected angiogenesis by inhibiting of VEGF expression, endothelial cell migration, and vascularization [64,69,70]. In addition, numerous clinical trials of proanthocyanidins have been performed for the treatment of various diseases in patients, and in healthy subjects and pregnant women [10,71,72]. These studies consistently show that proanthocyanidins are effective and safe, warranting their application to a variety of diseases. However, the efficacy of proanthocyanidins in the treatment of psoriasis has not yet been tested directly.

## Hypothesis for proanthocyanidins in the management of psoriasis

**Figure 2.**
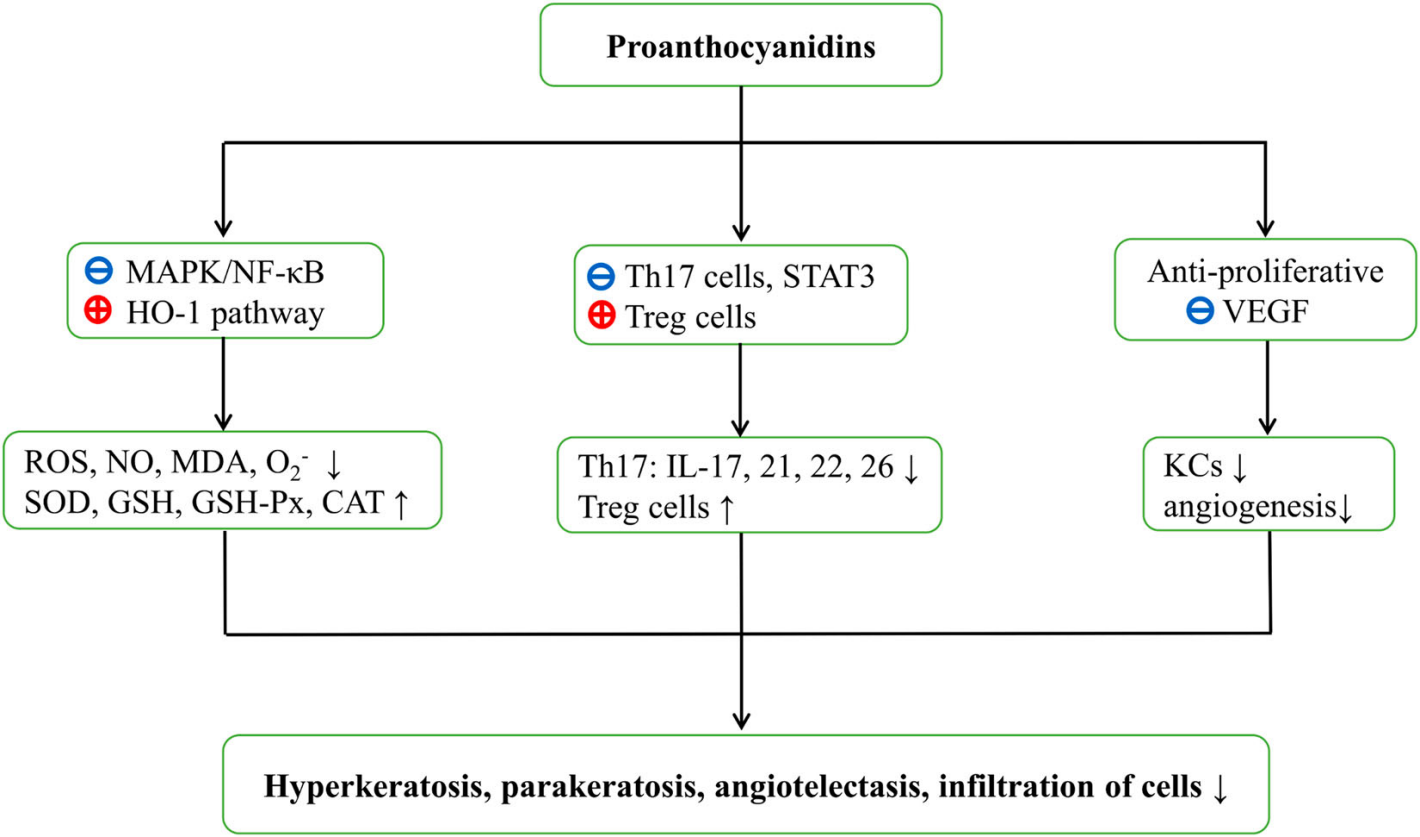
Hypothesized mechanisms of action of proanthocyanidins against psoriasis. Proanthocyanidins block MAPK/NF-κB signaling pathways and activate HO-1 expression. Oxidative stress parameters such as reactive oxygen species and malondialdehyde are then decreased with increasing antioxidant levels. Proanthocyanidins also reduce Th17 cell numbers and moderate the release of STAT3-dependent cytokines. Moreover, increased Treg cell numbers in the presence of proanthocyanidins may facilitate immunological tolerance. Furthermore, proanthocyanidins are anti-proliferative and may prevent VEGF expression. Ultimately, all these aspects are likely to contribute to the control of psoriasis. ROS, reactive oxygen species; NO, nitric oxide; MDA, malondialdehyde; ${\text{O}}_{2}^{-}$, superoxide radical; SOD, superoxide dismutase; GSH-PX, glutathione peroxidase; GSH, glutathione; CAT, catalase; STAT3, signal transducers and activators of transcription; Th, T helper; Treg cells, regulatory T cells; IL, interleukin; VEGF, vascular endothelial growth factor; KCs, keratinocytes.

Because OS insult and T-cell dysregulation are characterized pathogenic consequences of psoriasis, and proanthocyanidins have established antioxidant, anti-inflammatory, anti-angiogenic, anti-proliferative, and immunomodulatory properties, it is likely that proanthocyanidins will be of benefit to psoriasis patients. Here, we present lines of evidence for the potential of proanthocyanidins in the treatment of psoriasis as follows: (1) Psoriasis is a common immune-mediated inflammatory skin disorder that presents as keratinocyte hyperproliferation, epidermal hyperplasia, angiogenesis, and inflammatory cell infiltration [1,56]. (2) OS insult and immune inflammation have been associated with the pathogenesis of psoriasis [27–29]. (3) Elevated ROS and oxidant/antioxidant imbalances have been shown with increased Th17/Treg ratio in psoriatic lesions and in serum samples [24–26], and these conditions are known to trigger and sustain the progression of psoriasis [47–49]. (4) Numerous inflammatory mediators/cytokines (IL-17, IL-23, VEGF, TNF-α, TGF-β, and IFN-γ) and several signaling pathways (NF-κB, MAPK, and STAT3) are upregulated and activated in psoriatic tissues [22,42,48,49,51]. (5) Proanthocyanidins are natural extracts with no known side effects and antioxidant, anti-inflammatory, anti-angiogenic, anti-proliferative, and immunomodulatory activities [16,57–65]. (6) Proanthocyanidins ameliorate various OS-related diseases by scavenging ROS and MDA, by blocking MAPK/NF-κB signaling pathways, and by upregulating HO-1, CAT, SOD, and GSH-Px [14,57,58,68]. (7) Proanthocyanidins also decrease Th17/Treg ratios and expression levels of inflammatory cytokines and STAT3, and have been used to treat various immune-mediated diseases [15,59]. (8) Finally, proanthocyanidins slowed tumorigenesis by inhibiting cell proliferation, VEGF expression, and angiogenesis [16,62,64,69,70,73]. Hypothetical mechanisms of proanthocyanidins in the treatment of psoriasis are broadly summarized in terms of OS inhibition, mediation of proinflammatory signaling pathways, and regulation of T cells (Figure 2).

## Clinical significance

Psoriasis has long been a research focus in the field of dermatology. Despite the use of various drugs and physical therapies to control psoriasis, these strategies are limited to short-term use owing to their transient efficacy, high costs, and serious side effects. As natural active substances without side effects, proanthocyanidins are excellent candidates for the treatment of psoriasis, and their antioxidant, anti-inflammatory, anti-angiogenic, anti-proliferative, and immunomodulatory activities will likely ameliorate OS, Th17/Treg cell imbalances, keratinocyte over-proliferation, and angiogenesis. Finally, proanthocyanidins are safe for infants, pregnant women, and the elderly [72,74].

## Future research

**Figure 3.**
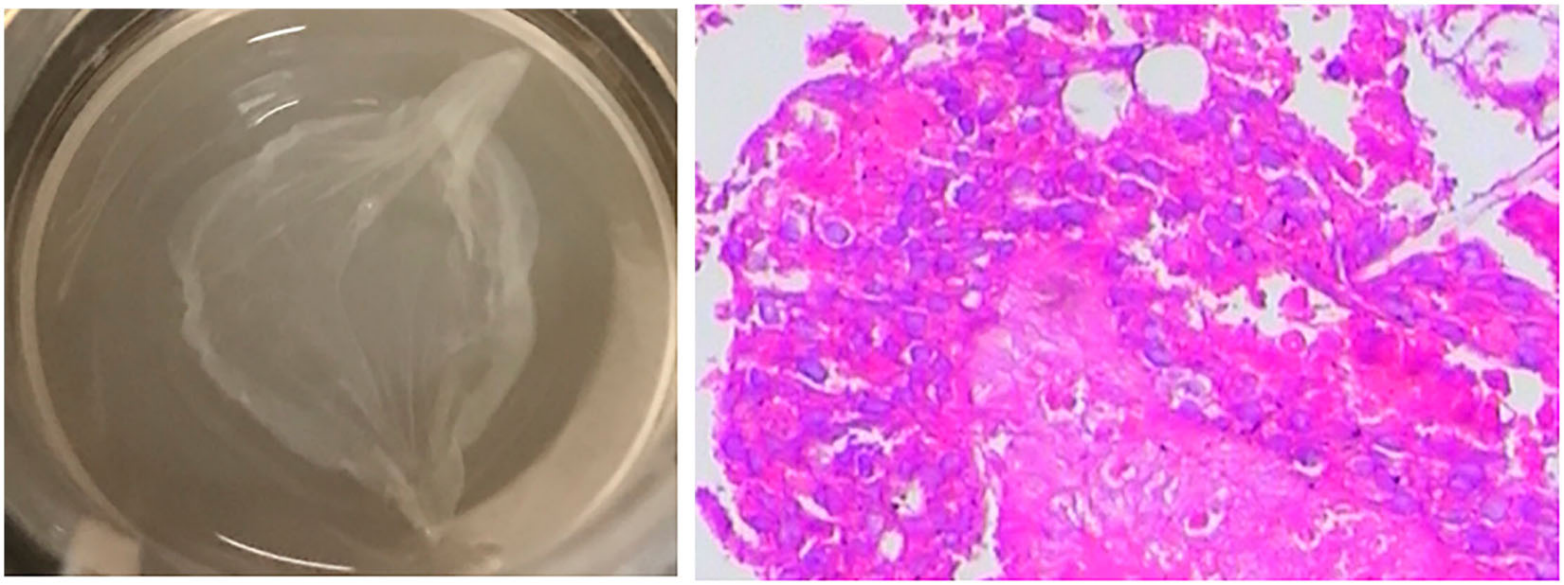
Previously, we established a psoriatic-like three-dimensional *in vitro* skin model. (A) Appearance of three-dimensional skin in our experiments; (B) histopathology of the three-dimensional skin model; hematoxylin and eosin staining (×200).

Future studies are required to monitor psoriasis related histopathological alterations such as hyperkeratosis, parakeratosis, angiotelectasis, microabscesses, and immune cell infiltration in the presence and absence of proanthocyanidins. Herein, we speculate that proanthocyanidins have potent therapeutic effects on psoriasis. To test this hypothesis and clarify the effects of proanthocyanidins in psoriasis patients, animal experiments are necessary to investigate the effects of dietary proanthocyanidins in well-established psoriatic mice models. Subsequent *in vitro* studies could be performed using psoriatic-like three-dimensional skin, as suggested in our previous study (Figure 3). Finally, placebo-control clinical trials may offer a scientific basis for further studies and clinical applications of proanthocyanidins.

## Conclusion

In summary, psoriasis is a common skin disease that negatively affects quality of life. It is widely regarded as a multifactorial condition involving OS aggression, T-cell dysregulation (Th17 and Tregs in particular), and genetic susceptibility (typical example being TNF-α gene polymorphism) as well as a complicated cytokine network [35]. By targeting these key points, psoriasis can be hoped to be cured. Now, a new generation of biologics, cytokine blockers targeting key cytokines or pathways, have shown positive efficacy for psoriasis in clinical trials [75]. Etanercept, a typical biologic for psoriasis, targets TNF-α (+489 GG and + 489 GA genotypes) and is effective in treating psoriasis, especially psoriasis arthropathica [41,76,77]. Ustekinumab, another novel biologic, a monoclonal antibody targeting the common p40 subunit, has an encouraging effect on psoriasis by inhibiting IL-12 and IL-23. Besides, psoriasis positively responds to the treatment involving IL-17 antibodies (e.g. secukinumab, ixekizumab, and brodalumab) that targets the IL-17 cytokine pathway to alleviate the inflammatory response [78–80]. Although biologics display improved effect on psoriasis, the treatment cost prevents patients from adopting the treatment. Thus, cost-effective therapies are required for psoriasis. Herein, we clarify the pathogenesis of psoriasis in terms of the properties and clinical applications of proanthocyanidins. The present cited studies suggest that proanthocyanidins are an ideal candidate for the management of psoriasis and can be used in combination with other drugs, such as anti-cytokine biologics. However, further *in vivo* and *in vitro* experiments are required to confirm improvements in psoriasis disease parameters following treatments with proanthocyanidins.
